# The Cherrattah

**Published:** 1828-12

**Authors:** Thomas Baker

**Affiliations:** Stamford-Street, Blackfriars


### THE CHERRATTAH.

*To the Editors of the London Medical and Physical Journal.*

Sirs,—It has long been a matter of surprise to me that the herb Cherrattah, which has been held, from time immemorial, in great estimation by the natives of Bengal and the European residents, especially the medical officers, as a very efficacious deobstruent and stomachic medicine, should not have been introduced into the practice of this country; especially as the variety of dyspepsia for which it is considered a specific, (accompanied with, and probably dependent on, sluggish or overloaded state of the liver,) is as prevalent in this country as in the East Indies.

It is said that the effects of the cherrattah are not, like stomachics in general, confined to the stomach, but extended to the abdominal viscera, particularly the liver, which it deterges, or (as Dr. Currie observes) emulges: and this I believe to be the case; for I have observed the feces, during its use, to be well charged with bile, and the complexion to become clear. Although not aperient, it evidently prevents an accumulation of feces of the lower portion of the intestinal canal, which, as a late writer observes, is a common cause of disorders of the stomach and head, and at the same time promotes digestion.

The medicinal virtues of the herb are imparted to boiling water, and the infusion, when properly made, is a very grateful bitter; but the natives prefer the decoction made by gently boiling half an ounce of the cut dried herb in a pint of water for about fifteen or twenty minutes. Of this decoction they take a small wine-glassful two or three times a day. The extract, which also contains the virtues of the herb in great perfection, is taken in the form of pills.

It is likewise given by the Indian practitioners in cases of pulmonary consumption and scrofula; but of its effects in the former malady I cannot speak from experience. In the latter malady I have frequently witnessed its salutary operation. Dr. Flemming speaks highly of the cherrattah as a tonic medicine. Dr. James Johnson, in his work on Tropical Diseases, also gives it a high character; and Mr. Addison, in his Treatise on the Malvern Water, says that, from the very beneficial effects it had on himself, he looks upon it as a valuable addition to the class of stomachic medicines.

I am, gentlemen,
Your very obedient servant,
THOMAS BAKER.

*Stamford-street, Blackfriars; October* 1828.

I have enclosed a small quantity of the herb, and of the extract of it made from a cold infusion.

